# A20 Haploinsufficiency in a Chinese Patient With Intestinal Behcet's Disease-Like Symptoms: A Case Report

**DOI:** 10.3389/fimmu.2020.01414

**Published:** 2020-07-03

**Authors:** Yu Chen, Huanjun Huang, Yao He, Minhu Chen, Ursula Seidler, De'an Tian, Fang Xiao

**Affiliations:** ^1^Department of Gastroenterology, Tongji Hospital of Tongji Medical College, Huazhong University of Science and Technology, Wuhan, China; ^2^Department of Gastroenterology, First Affiliated Hospital of Sun Yat-sen University, Guangzhou, China; ^3^Department of Gastroenterology of Hannover Medical School, Hanover, Germany

**Keywords:** haploinsufficiency of A20, intestinal Behçet's disease, autoimmune disorder, TNFAIP3, gene mutation, whole exome sequencing, autosomal-dominant-inherited disease, expressivity

## Abstract

**Objective:** Intestinal Behcet's disease (iBD) is an autoimmune disorder diagnosed by typical intestinal ulcers and systemic Behcet's disease (BD) manifestations. Haploinsufficiency of A20 (HA20) is a recently described autoinflammatory disease with a phenotype resembling BD, caused by heterozygous loss-of-function mutations in *TNFAIP3* gene (encoding A20).

**Methods:** We described a 29-year-old female with iBD-like symptoms including relapsing ulceration of intestinal anastomosis, recurrent oral ulcers and vasculitis in extremities. Due to the atypical intestinal ulcers with long segmental involvement and intestinal obstruction, whole exome sequencing (WES) was performed to screen for the underlying genetic defect and the identified gene was confirmed by Sanger sequencing. The expression levels of A20 was evaluated by Western blot. Sanger sequencing and Western blot were also performed in the patient's family members.

**Results:** A heterozygous mutation of *TNFAIP3* (c.305A>G, p. Asn 102 Ser) was identified in the patient. The identical *TNFAIP3* mutation was also found in her father and brother who had suffered from recurrent oral ulcers since childhood. Functional experiments revealed that the expression of A20 was decreased in the peripheral blood mononuclear cells of the patient and her family members who carried the TNFAIP3 mutation.

**Conclusion:** We described a Chinese patient with a novel heterozygous mutation in *TNFAIP3* who developed iBD-like symptoms. We proposed that the *TNFAIP3* heterozygous mutation (c.305A>G, p. Asn 102 Ser) with an insufficient expression of A20 may be associated with the iBD phenotype in patients.

## Introduction

Behcet's disease (BD) is an autoimmune disease with a polygenic background and is mainly identified by recurrent oral aphthous ulcers, genital ulcers, and ocular, vascular, and gastrointestinal lesions (1). BD patients with predominantly gastrointestinal symptoms and intestinal ulceration may be diagnosed with intestinal Behcet's disease (iBD) (2). The diagnosis of iBD is dependent on the presence of typical intestinal ulcers and clinical manifestations (2). The typical intestinal ulcer of iBD is defined as less than five ulcers that are oval in shape, deep with discrete borders, and located in the ileocecal area (2). Haploinsufficiency of A20 (HA20) is a newly described autoimmune disorder with one of the various phenotypes resembling BD (3). HA20 is caused by heterozygous loss-of-function mutations of the *TNF Alpha Induced Protein 3 (TNFAIP3)* gene encoding A20 and the diagnosis of HA20 mainly depends on genetic analysis (4). Here, we reported a HA20 patient with intestinal Behcet's disease-like symptoms, including relapsing ulceration of intestinal anastomosis, recurrent oral ulcers and vasculitis in extremities.

## Case Report

**Figure 1:**
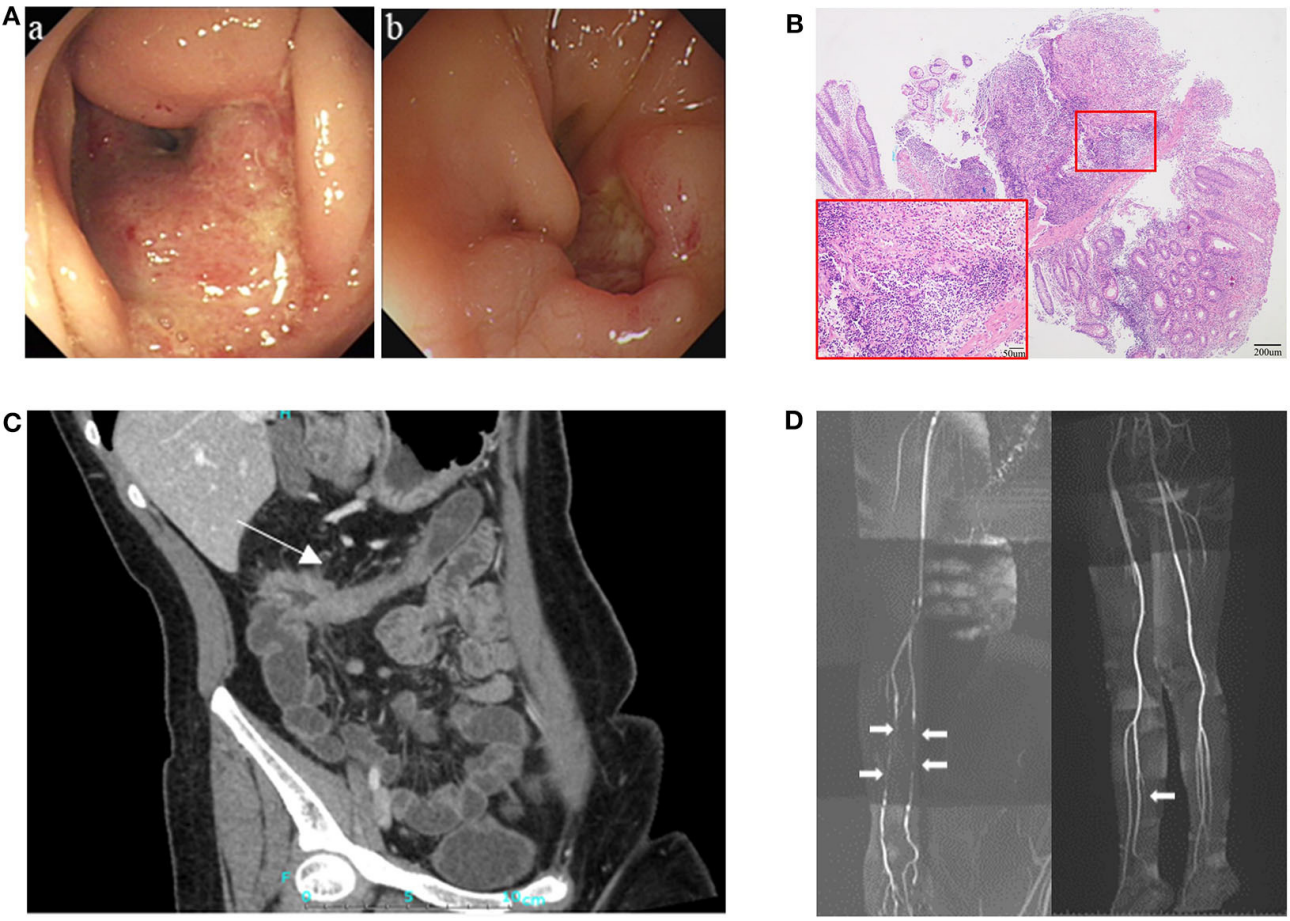
Clinical presentation of the patient. **(A)** Endoscopy showed ulcers and stricture in the ascending colon in August 2010 (a), and relapsing ulceration of intestinal anastomosis and stricture in transverse colon 8 years after right hemicolectomy (b). **(B)** Pathological examination showed intestinal transmural inflammation with an increased number of inflammatory cells infiltration, loss of crypt and hyperplasia of fibrous tissue. **(C)** CT enterographic (CTE) image demonstrated thickening of the wall and stricture in the colon-hepatic curvature and the proximal transverse colon (arrow). **(D)** Magnetic resonance angiography (MRA) showed multiple arteritis stenosis in upper and lower limbs (white arrows).

The patient was a 29-year-old female who had suffered from recurrent oral ulcers, abdominal pain and diarrhea since the age of 15 years. Recurrent ascending colonic ulcers and anastomotic ulcers along with intestinal obstruction were observed prior to and for 8 years after right hemicolectomy surgery (Figures 1A–C), accompanied by vasculitis in extremities (Figure 1D). Laboratory data showed elevated C-reactive protein (19.6 mg/L; normal range (NR) <5 mg/L) and erythrocyte sedimentation rate (25 mm/h; NR<20 mm/h), along with a low titer of the antinuclear antibody (1:100). Serum IgG and IgM were within the normal range but IgA levels were low (IgG 19.3–25.8g/L, IgM 0.73–0.92 g/L, IgA <0.07g/L). The patient responded to a glucocorticoid and thalidomide in 3 months with reduced frequency of diarrhea and less severe abdominal pain. The patient's IgA increased but did not reach normal levels after treatment. Subsequent inquiries into the patient's family history revealed that her father suffered from recurrent oral ulcers when he was young, and her brother had suffered from recurrent fever, oral ulcers and erythema nodosum-like lesions in the skin since he was 4 years old. The level of serum immunoglobulins in the father and brother were in the normal ranges. Because of the mild and non-specific symptoms, they accepted treatment of only antimicrobial mouthwash and dental ulcer paste instead of immunomodulators.

## Genetic Analysis

**Figure 2:**
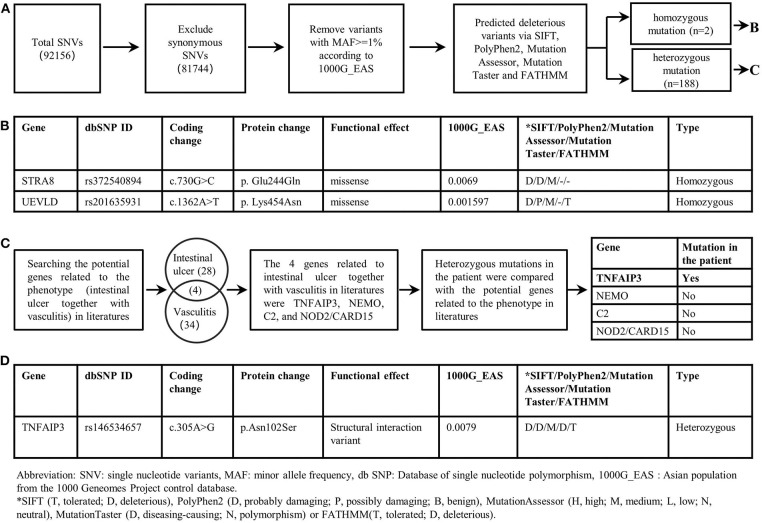
Filtering strategies for candidate disease-causing SNVs in the patient. **(A)** Screening for predicted deleterious variants in the patient, including homozygous mutations and heterozygous mutations. **(B)** The genetic information of homozygous mutations identified in the patient. STRA8 and UEVLD mutations were not reported to be associated with intestinal ulcers or vasculitis. **(C)** The procedure of screening candidate variants based on the phenotype of the patient. Genes likely related to intestinal ulcer together with vasculitis were screened out by searching in literatures. And four genes including TNFAIP3, NEMO, C2, NOD2/CARD15 were reported to be related to intestinal ulcer together with vasculitis. By comparing the heterozygous gene mutations in the patient with the above four genes, the TNFAIP3 heterozygous mutation was identified as the candidate disease-causing mutation in the proband. **(D)** The genetic information of the TNFAIP3 gene mutation in the patient.

**Figure 3:**
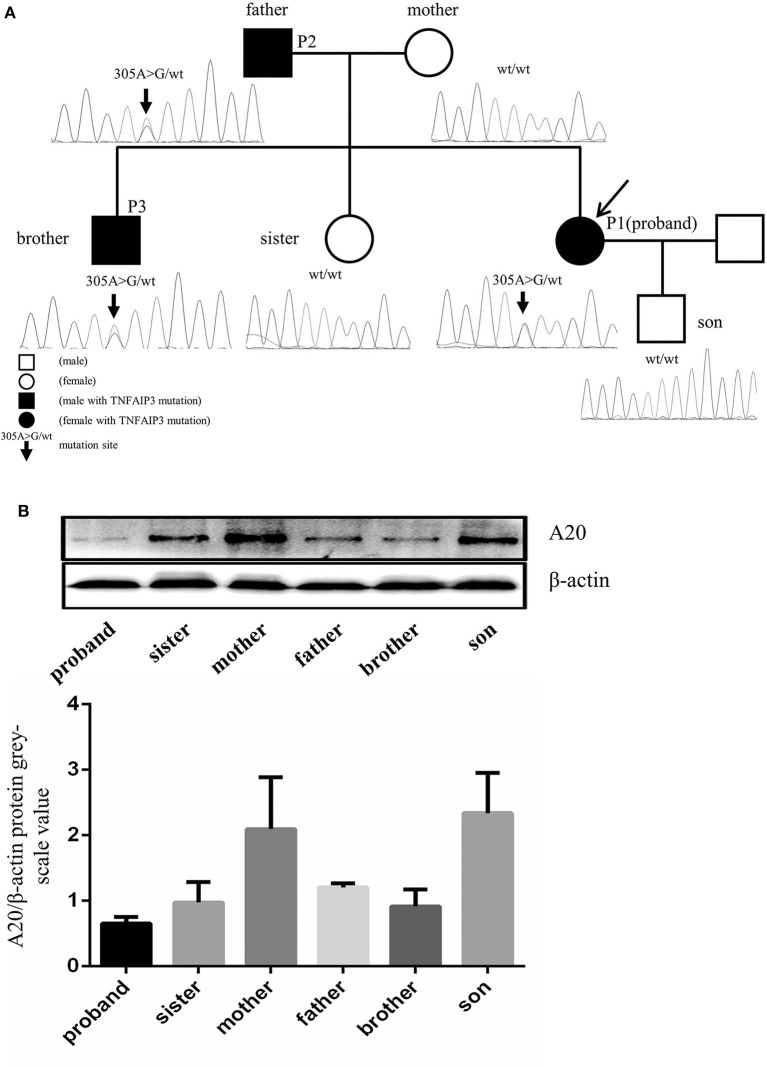
TNFAIP3 mutation and the A20 expression in PBMC of the proband and her family members. **(A)** Sequencing analysis of the TNFAIP3 gene among the proband and her family members revealed a heterozygous mutation (c.305A>G, p. Asn 102 Ser) in the proband, her father and her brother. **(B)** Western blot analysis showed that TNFAIP3 expression in PBMC from the patient, her father, brother and sister were decreased as compared to other family members without TNFAIP3 mutation. The bar graph represented semi-quantification of Western blot analysis from 3 independent experiments.

Whole exome sequencing (WES) was performed on the patient. In our study, single nucleotide variants (SNVs) with minor allele frequency (MAF)<0.01 in the Asian population of the 1,000 Genomes Project (1000G_EAS) were supposed to be potential disease-causing mutations; the distribution of BD shows a racial difference with a higher incidence in eastern Asian populations along the “Silk Road” (5). The 1,000 Genomes Project (1000G) facilitates genetic variation analysis within and between races by providing a detailed view of variation across several races which includes a Chinese group (6). The MAF of screened SNVs were also investigated in other common genome sequencing databases. Candidate disease-causing variants of the patient were screened based on the reported gene mutations associated with intestinal ulcer and vasculitis. The screening of a causal variant followed a standard procedure (7). The WES revealed two homozygous deleterious mutations of *stimulated by retinoic acid gene 8 (STRA8)* and *UEV and lactate/malate dehyrogenase domains (UEVLD*), but these were not associated with the phenotype of intestinal ulcer and vasculitis according to the literature. A heterozygous deleterious mutation of TNFAIP3 related to intestinal ulcer and vasculitis was identified by the WES (c.305A>G, p. Asn 102 Ser) (MAF=0.0079 in 1000G_EAS, MAF=0.0130 in 1000G_ALL, MAF=0.0030 in GnomAD, MAF=0.0135 in GnomAD_EAS, MAF=0.01734 in ExAC). The details of the WES results and variant filtering procedure are given in Figures 2A–D. Sanger sequencing was performed for the patient's whole genealogy and the identical mutation of *TNFAIP3* was found in her father and younger brother (Figure 3A).

## Expression of A20 in the Patient and Her Family

Protein was extracted from the peripheral blood mononuclear cells (PBMCs) of the patient and her family members and the expression of A20 was evaluated by Western blot. The levels of A20 were decreased in the PBMCs of the patient and her family members who carried the identified mutation (Figure 3B). To our surprise, her pregnant sister who lacked the *TNFAIP3* mutation also showed a lower level of A20 expression compared to other family members without the *TNFAIP3* mutation.

## Discussion

A20 plays an important role in regulating immunity by inhibiting NF-κB signaling, activation of NLRP3 inflammasome, and apoptosis (8, 9). Haploinsufficiency of A20 is characterized by an upregulated inflammatory reaction and manifests symptoms that resemble many autoimmune diseases, including BD, rheumatoid arthritis (RA) and systemic lupus erythematosus (SLE) (3). The patient in our report presented with iBD-like symptoms, including relapsing ulceration of intestinal anastomosis, recurrent oral ulcers and vasculitis in extremities; although, the patient's intestinal ulcer was atypical due to the long segment involvement in the intestine and incomplete intestinal obstruction. WES and Sanger sequencing identified a novel *TNFAIP3* mutation in the patient that has not been previously reported in HA20. *TNFAIP3* mutations may lead to a reduced transcription and instability of mutant proteins (4, 7, 9). This mutation causes decreased A20 expression and results in immune dysregulation with increased NF-κB activity in response to TNF-α (10); it is also proposed as a risk factor for autoimmune disorders (11, 12). Additionally, *TNFAIP3* is reported to be linked with susceptibility to BD (4, 13, 14). Intestinal Behcet's disease is considered a polygenic disease with a strong genetic background, but its diagnosis is still based on typical intestinal ulcers and manifestations of systemic BD (2). The homozygous mutations of STRA8 and UEVLD revealed through WES were not considered the disease-causing genes because they were likely non-contributory to the disease phenotype of intestinal ulcer and vasculitis previously documented in the literature. Our case reported that the *TNFAIP3* gene is also associated with iBD-like symptoms, suggesting that for patients with iBD-like symptoms that lack typical intestinal ulcers, genetic screening for TNFAIP3 could be considered.

HA20 is an autosomal-dominant-inherited disease that has been described with considerable variation in its expressivity (15). Consistent with the features of an autosomal-dominant-inherited disease, the three members in the reported family that carried the identical *TNFAIP3* mutation showed varying degrees of symptoms. When an autosomal-dominant-inherited disease occurs in a pedigree without apparent familial hereditary traits, it is an option to identify the genetic mutation by initially screening for a causal mutation in the proband by WES and then further identifying the mutation in pedigrees by Sanger sequencing. When we analyze a genetic disease with a specific difference, deleterious variants should be screened for in an appropriate genome sequencing database in accordance with the relevant population.

A reproducible decrease in TNFAIP3 expression was also found in the patient's pregnant sister who lacked the *TNFAIP3* mutation. This can be explained by the fact that some factors other than TNFAIP3 mutation can influence the expression of A20, such as hyperglycemia, oxidative stress, and high levels of 17β-estradiol (16–18). The decreased level of A20 in the patient's sister may have resulted from these other factors rather than a *TNFAIP3* mutation.

In conclusion, we presented a HA20 patient with iBD-like symptoms and identified a novel *TNFAIP3* heterozygous mutation locus (c.305A>G, p. Asn 102 Ser) in HA20. Looking forward, genetic screening for TNFAIP3 could be considered for patients with iBD-like symptoms.

## Data Availability Statement

The original contributions presented in the study are included in the article/supplementary material, further inquiries can be directed to the corresponding author/s.

## Ethics Statement

Informed consent and blood sample collection were obtained from the patient for the publication of this case report. Approval for the study was obtained from the Ethical Committee of Tongji Hospital, Tongji Medical College, Huazhong University of Science and Technology. Informed consent was provided according to the Declaration of Helsinki.

## Author Contributions

FX and YC: study concept and design, acquisition, analysis and interpretation of data, and drafting of the manuscript. HH, FX, YH, and DT: collection, analysis, and interpretation of clinical data. US and MC: a critical review of the manuscript. All the authors approved the final draft of the manuscript submitted for publication.

## Conflict of Interest

The authors declare that the research was conducted in the absence of any commercial or financial relationships that could be construed as a potential conflict of interest.
